# Correlates of feeding difficulties among children of Chinese transgender parents

**DOI:** 10.1080/26895269.2024.2317401

**Published:** 2024-02-25

**Authors:** Haibing Yang, Xiaona Na, Yuefeng Tan, Menglu Xi, Yucheng Yang, Ai Zhao

**Affiliations:** Vanke School of Public Health, Tsinghua University, Beijing, China

**Keywords:** Chinese, feeding difficulties, parenthood, transgender

## Abstract

**Background:**

Ensuring proper early feeding for young children is crucial, as encountering feeding difficulties (FD) during this stage can give rise to a cascade of health problems, the repercussions of which may endure into late childhood and adolescence. Children raised by transgender parents may be at risk of encountering FD, however, there is no research conducted on Chinese transgender families.

**Methods:**

We designed a cross-sectional survey in which the rate of FD and its influencing factors were investigated among transgender parents in China. A total of 446 Chinese transgender parents (average age 30.39 years) were included in the analysis. Logistic regression models were applied to investigate the influencing factors of FD among children of transgender parents. We also established structural equation modeling (SEM) to explore the possible pathways among these factors and FD.

**Results:**

The rate of FD in children of Chinese transgender parents is 55.4%, with 34.5% having severe FD. Coming out after having a child (AOR = 2.26, 95%CI = 1.33 ∼ 3.91), family violence (AOR = 1.06, 95%CI = 1.04 ∼ 1.09), partner violence (AOR = 1.11, 95%CI = 1.08 ∼ 1.15), no feeding education (accepting feeding education: AOR = 0.43, 95%CI = 0.25 ∼ 0.74), being discriminated during seeking of childbearing health care (AOR = 1.99, 95%CI = 1.3 ∼ 3.05), and poor relationship with partner (fair: AOR = 0.09, 95%CI = 0.03 ∼ 0.22; good: AOR = 0.06, 95%CI = 0.02 ∼ 0.15) are significantly associated with higher FD. Furthermore, through the pathway analysis, the indirect effects of education level (β=−0.151), feeding education (β = 0.145), and relationship with partner (β=-0.196) on FD are observed.

**Conclusions:**

Children of Chinese transgender parents showed a high FD rate. It is crucial to help build a better family and social environment for transgender families to reduce the FD and improve children’s and adolescents’ health.

## Introduction

Feeding difficulties (FD) can be broadly defined as a range of feeding problems, including picky and highly selective eating, protracted mealtimes, crying or agitation during feeding times, food refusal, pushing or throwing food away, chaotic and upsetting mealtimes, inadequate independent feeding, using distractions to increase intake, or neglecting to introduce advanced textures (Lewinsohn et al., 2005; Maslin et al., 2015). Feeding problems in children are common, occurring in 20% to 50% of children who develop according to typical trajectories (Milano et al., 2019). Feeding problems in early childhood, if unaddressed, might track into later adolescence and even adulthood (Powell et al., 2018). A small problem persisting for a prolonged time may also significantly impact a child’s nutritional status, physical growth, cognitive development, and behavioural and emotional disorders (Sanchez et al., 2015; Volkert & Piazza, 2012). FD can also lead to parental stress, anxiety, and depression, as well as fear of social stigmatization due to unconventional feeding practices (Sharp et al., 2017). Research into the causes and correlates of FD is critical for conducting interventions and prevention programs to improve the eating behaviours of children and adolescents.

Currently, it is generally accepted that FD are essentially biopsychosocial issues (Goday et al., 2019), because both physiological and psychosocial factors contribute to their initiation and maintenance. Possible explanations include medical factors (e.g. developmental and neurological disabilities), infantile temperament and psychological factors, parental feeding styles, parent-child interactions, and socio-environmental factors (Ramsay et al., 2011; Ren et al., 2021). However, the influencing factors of FD likely vary greatly across different cultural contexts and populations (Ren et al., 2021).

According to a literature review of parenting intentions in transgender communities, a majority of transgender adults report a desire to have children (54%-82%), and about half of transgender adolescents have a desire to have children (48.7%-67%) (Stolk et al., 2023). For some transgender individuals, interventions such as hormone treatments and gender-affirming surgeries are medically necessary to conform one’s body to one’s gender (Clark, 2021). These treatments can have irreversible effects on fertility, especially gender affirming surgery.

It is reassuring to know that an increasing number of healthcare providers and stakeholders have begun to respect avenues for family creation that are normative within trans communities (Lampe et al., 2019). Some countries now allow sexual and gender minorities to adopt children, or support transgender individuals to retain their fertility during gender-affirming treatment (Payne & Erbenius, 2018), bringing more options for transgender people to become parents. In mainland China, however, transgender people’s options for starting a family remain limited. In addition, sterilization is a prerequisite for gender-affirming treatment in mainland China (Jiang et al., 2014), unmarried individuals may not access assisted reproductive technology in most regions of China, and surrogacy is banned (UNDP & China Women’s University, 2018), which may bring more difficulties for transgender people to have and raise a child. In addition, empirical studies have consistently shown that many transgender individuals are already parents (Carone et al., 2021; Tasker & Gato, 2020).

In contrast to cisgender parenthood, transgender parenthood arises in the context of systemic barriers, widespread discrimination, social stigma, and the continued pathologizing of transgender people (Carone et al., 2021). In mainland China, transgender individuals face insufficient social support, and many of their parents, influenced by traditional attitudes, are still unable to accept them, even forcing them to "carry on the family line" (Xie et al., 2021). Previous studies suggested that transgender parents show a high need to demonstrate their ability and adequacy to fulfill their parental role (De Castro-Peraza et al., 2019). Furthermore, previous research revealed that transgender parents need to play multiple contradictory roles in trying to integrate their parenting and gender identities (Hafford-Letchfield et al., 2019; Haines et al., 2014). In this case, the intention and effort of transgender people to become parents, as well as multiple aspects of their adaptation to parenthood, may be undermined. This could lead to an increased risk of FD for their children, as previous research has shown that FD in children are related to the feeding behaviours of parents (Rogers et al., 2018).

The existing quantitative literature on parenting experiences of transgender people is limited in China. The focus of previous research has mainly been on how to conceive (Payne & Erbenius, 2018), pregnancy (Brandt et al., 2019), and lactation (Yang et al., 2023). The research on the subsequent parenting process mainly focuses on the parent-child relationship and psychopathology in school-aged children or adolescents (Dierckx et al., 2016; Hafford-Letchfield et al., 2019; White & Ettner, 2007). To our knowledge, there is no data to depict FD among preschool children in Chinese transgender families. To help transgender parents successfully enter the world of parenting and further prevent the life-long adverse effects caused by FD for their children, there is still much to be learned about the parenting performance and associated factors of transgender parents (Pfeffer & Jones, 2020).

In this study, we conducted a large-scale cross-sectional study of transgender parents living in China, investigating the presence of FD in their children, exploring the relevant factors, and making possible recommendations to improve the situation.

## Methods

### Participants

Between January 2022 and February 2022, an online cross-sectional survey of transgender parents was conducted in China. Full details of the study procedures and sample have been previously published (Yang et al., 2023). Briefly, we consulted experienced maternal and child health professionals and researchers, experts with research experience related to transgender population, and volunteers from transgender communities, to develop the survey materials. Participants were recruited using a sampling strategy that combined convenience, respondent-driven, and snowball sampling, all of which have been shown to be effective in recruiting gender and sexual minorities (Chen et al., 2019; Parmley et al., 2022; Westafer et al., 2022). First, we used social media platforms to deliver an online survey to transgender communities and recruited the first wave of participants. Then, the involved participants were invited to help identify further recruits and distribute the questionnaire to their transgender friends and acquaintances (transgender parents who were from the same family were prevented from being invited *via* a notice shown in the invitation letter). E-questionnaires and online recruitment advertisements were distributed through social media platforms (Wenjuanxing Tech Co., Ltd., Changsha, China). All participants remained anonymous, and before participation, informed consent was obtained by clicking “agree to participate in this study”.

The inclusion criteria of this study were: (1) assigned gender that did not coincide with self-identified gender; (2) having or used to have at least one child in a biological or legal sense; and (3) currently living in China and being able to read Chinese. The exclusion criteria were (1) having repetitive IP addresses, (2) spending less than 5 min to complete the survey; (3) choosing the same answer in “What is your assigned gender?” and “What is your gender identity?”. Participants who met the inclusion criteria received a monetary incentive (RMB 10-15) for completing the survey. To ensure the quality of the survey, three attention-check questions were used. If a participant chose the wrong option in any of these questions, this indicated poor data quality and was therefore excluded from this study. After inclusion and exclusion, we reached a total of 647 participants, and only 466 participants with children younger than 6 years of age were included because the feeding scale used in this study was applied to subjects younger than 6 years of age (Ramsay et al., 2011). The sample source included 30 provinces in mainland China. The study was conducted in accordance with the Helsinki Declaration and was approved by the Ethics Committee of Tsinghua University (NO 20210161).

### Data collection

Data were collected through an electronic questionnaire that was reviewed and tested by experts in the related fields and transgender volunteers. The questionnaire consisted of five parts: sociodemographic characteristics, transgender-related factors, family social environment factors, feeding-related factors, and The Montreal Children’s Hospital Feeding Scale (MCH-FS). We collected information on participants’ transgender-related and family social environment characteristics by asking questions such as “Have you come out before having this child?”; “Have you had hormonotherapy (gender-affirming hormone intervention) before having this child?”; “Have you been discriminated against during seeking childbearing (such as when health providers didn’t respect my gender identity, showed prejudice, etc.)?”, and so on. The details of the questions on transgender-related and family social environment characteristics could be found in our previous studies (Yang et al., 2023). Regarding feeding-related factors, the questions were self-designed with reference to previous studies (Goday et al., 2019; Qiu et al., 2021; Van Dijk et al., 2016) including: “Who is the child’s primary caregiver?”; “Who is the child’s feeding decision maker?”; “Can you feed your child according to your child’s hunger signal?”; “Do you give verbal praise and emotional communication while feeding?”; “Does your child have a fixed feeding place/fixed feeding time/fixed caregiver?”; and “Does your child eat independently?”.

The validated Chinese version of the MCH-FS (Dai et al., 2011; Ren et al., 2021) was used in this study to evaluate the extent of children’s FD. MCH-FS is a brief screening tool for identifying feeding problems in targeted children six months to six years of age (Ramsay et al., 2011). The scale contains 14 items, and each item is rated on a seven-point Likert scale. Seven items were scored from the negative to the positive direction, and the others were in the reverse direction. The total score (raw score, range 14–98) was calculated by summing up the scores for all 14 items after reversing the scores of the seven items whose scales were from negative to positive (seven minus the raw score). The crude scores were then transformed into T-scores by the Logit transformation method. A T-score of 60 points or less means no FD; a T-score higher than 60 indicates risk for FD. In a previous MCH-FS Chinese standardization study, the MCH-FS could be classified into four dimensions after principal component analysis and have good structural validity (Dai et al., 2011). The four dimensions are: parental concern about feeding (PCF); oral motor function of infants (OMFI); feeding behaviour of parents (FBP); and eating behaviour of infants (EBI). The Cronbach coefficient α for the whole scale was 0.941 in this study, and 0.719, 0.854, 0.865, and 0.777 for the four dimensions, respectively, showing good reliabilities.

### Data analysis

The data were analysed using R statistical software version 4.1.3. Unpaired t-test and nonparametric test (Kruskal-Wallis test) were used to compare different continuous variables among the different FD levels after the normality test (using Shapiro-Wilk test) and homogeneity of variance test (using Bartlett test). The chi-square test was used to examine differences in categorical variables across FD levels. Logistic regression analysis with and without adjusting confounders was applied to explore the association between different factors and FD. Socio-demographic variables and feeding-related variables that were significantly associated with FD in bivariate analyses were added to adjusted regression models as covariates. We also did subgroup analyses in different gender identity groups (transgender woman and transgender man). We also analysed associations between factors and and different dimensions of MCH-FS. Variables that were significantly associated with FD in bivariate analyses of different groups were added to adjusted regression models, respectively. In the analyses of different dimensions of MCH-FS, we established generalized additive models because the scores of each dimension are not normally distributed. Structural equation modeling (SEM) was established to find possible pathways for how sociodemographic, feeding-related, transgender-related, and family environment variables impact CFPs using a weighted least-squares mean- and variance-adjusted (WLSMV) estimator. P values were 2-sided, and statistical significance was set at less than 0.05, and odds ratios (ORs) and 95% confidence interval (CIs) were used to report the results of logistic analyses.

## Results

### Individual item and total scores of the MCH-FS

**Figure 1.**
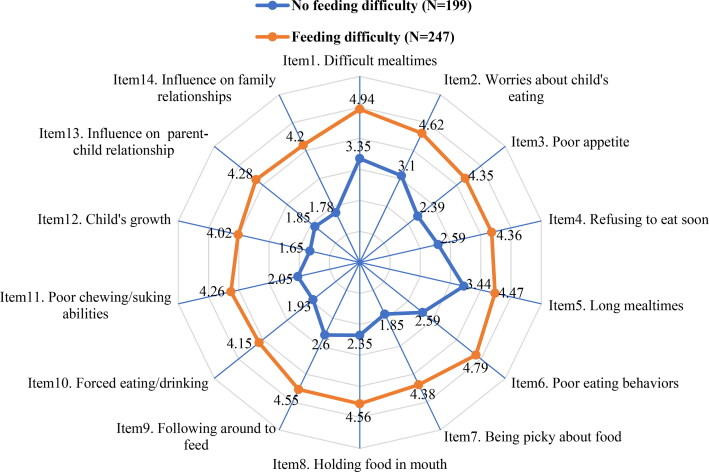
Mean score of the 14 items of MCH-FS based on classification of feeding difficulty.

Data of 446 responses was used in this study. The mean of individual item scores of the MCH-FS are shown in Figure 1. Of all the 14 items, the highest-scoring item was item 1 (“How do you find mealtimes with your child?”), and the lowest was item 12 (“How do you find your child’s growth?”). The mean total score of MCH-FS was 49.26 ± 16.80, and the mean T-score of MCH-FS was 63.18 ± 13.21.

The average age of the participants was 30.4 years. Among them, 252 (56.5%) self-identified as transgender women, 178 (39.9%) self-identified as transgender men, and 31 (7.0%) identified themselves as other (genderqueer or non-binary). Table 1 shows the differences in sociodemographic characteristics, feeding-related characteristics, transgender-related characteristics and family environment characteristics among parents whose children have different FD levels. A total of 55.4% (247/446) of all children were identified with FD. As shown in Table 1, among transgender parents with lower education levels (*p* < 0.001) or lower family annual incomes (*p* < 0.001), their children were more likely to have FD.

**Table 1.** Bivariate analyses of socio-demographic characteristics, feeding-related factors, transgender-related and family environment factors with feeding difficulties among children of transgender parents.

	Coding	No FD (*N* = 199)	FD (*N* = 247)	P
		(T score ≤ 70)	(T score > 70)	
Socio-demographic variables				
Age (mean (SD)):		30.17 (4.41)	30.57 (5.76)	0.409
Education level (%):				<0.001
Middle/high/technology/vocational school	1	15 (7.5)	54 (21.9)	
College/University (undergraduate)	2	160 (80.4)	182 (73.7)	
Postgraduate (Master/Ph.D.)	3	24 (12.1)	11 (4.5)	
Assigned sex (%):				0.686
Male	1	117 (58.8)	151 (61.1)	
Female	2	82 (41.2)	96 (38.9)	
Gender identity (%):				0.656
Transgender woman	1	109 (59.6)	143 (61.6)	
Transgender man	2	74 (40.4)	89 (38.4)	
Other	3	16 (8.0)	15 (6.1)	
Family type (%):				0.132
Extended family	1	84 (42.2)	106 (42.9)	
Nuclear Family	2	88 (44.2)	92 (37.2)	
Adoptive/reconstituted family	3	16 (8.0)	21 (8.5)	
Single parent or other	4	11 (5.5)	28 (11.3)	
Family annual income (%):				<0.001
<50k	1	28 (14.1)	62 (25.2)	
50–100k	2	33 (16.6)	60 (24.4)	
100–200k	3	66 (33.2)	68 (27.6)	
> =200k	4	72 (36.2)	56 (22.8)	
Reported gender of child (%):				0.803
Boy	1	111 (58.4)	143 (60.1)	
Girl	2	79 (41.6)	95 (39.9)	
Feeding-related variables				
Primary caregiver (%):				0.859
Parents	1	160 (80.8)	196 (79.7)	
Grandparents	2	38 (19.2)	50 (20.3)	
Feeding decision maker (%):				0.124
Parents	1	189 (95.0)	224 (90.7)	
Grandparents	2	10 (5.0)	23 (9.3)	
Feeding according to the infant’s hunger signal (%):				0.138
No	1	47 (23.6)	75 (30.4)	
Yes	2	152 (76.4)	172 (69.6)	
Verbal praise and emotional communication (%):				0.316
No	1	35 (17.6)	54 (21.9)	
Yes	2	164 (82.4)	193 (78.1)	
Fixed feeding place (%):				<0.001
No	1	68 (34.3)	126 (51.6)	
Yes	2	130 (65.7)	118 (48.4)	
Fixed feeding time (%):				0.001
No	1	49 (24.9)	99 (40.9)	
Yes	2	148 (75.1)	143 (59.1)	
Fixed caregiver (%):				0.003
No	1	24 (12.1)	57 (23.7)	
Yes	2	175 (87.9)	184 (76.3)	
Feeding at quiet place (%):				<0.001
No	1	28 (14.3)	82 (34.0)	
Yes	2	168 (85.7)	159 (66.0)	
Feeding method (%):				0.109
Feed by others	1	121 (60.8)	138 (55.9)	
Independently	2	22 (11.1)	45 (18.2)	
Mixed	3	56 (28.1)	64 (25.9)	
Transgender-related and family environment factors				
Time of coming out (%):				0.003
Before the coming of this child	1	132 (66.3)	135 (54.7)	
After the coming of this child	2	30 (15.1)	71 (28.7)	
Not yet	3	37 (18.6)	41 (16.6)	
Hormonotherapy (%):				0.416
Before pregnancy	1	51 (49.5)	73 (55.7)	
After lactation period or never	2	52 (50.5)	58 (44.3)	
Gender Dysphoria (mean (SD)):		42.86 (8.49)	42.92 (8.16)	0.947
Family violence (mean (SD)):		20.48 (8.10)	27.96 (13.86)	<0.001
Partner violence (mean (SD)):		16.21 (5.56)	23.27 (11.52)	<0.001
Family formation method (%):				0.167
Sexual intercourse	1	146 (73.4)	195 (78.9)	
Artificial insemination	2	16 (8.0)	24 (9.7)	
Surrogacy	3	20 (10.1)	17 (6.9)	
Adoption or other	4	17 (8.5)	11 (4.5)	
Feeding Education (%):				0.022
No	1	30 (15.1)	60 (24.3)	
Yes	2	169 (84.9)	187 (75.7)	
Discrimination during seeking of childbearing health care (%):				<0.001
No	1	118 (59.3)	104 (42.1)	
Yes	2	81 (40.7)	143 (57.9)	
Relationship with partner (%):				<0.001
Poor	1	6 (3.0)	68 (27.5)	
Fair	2	60 (30.6)	77 (31.6)	
Good	3	130 (66.3)	99 (40.6)	

### Transgender-related, feeding-related and family environment characteristics of participants and their association with FD

As Table 1 shows, FD were more likely to exist among children of participants who did not come out before the coming of this child (*p* = 0.003), used to suffer from family violence (*p* < 0.001) or partner violence (*p* < 0.001), had not received feeding education (*p* = 0.022), had experienced discrimination during seeking childbearing health care (*p* < 0.001), or had a worse relationship with their partners (*p* < 0.001). Furthermore, children who had a fixed feeding place (*p* < 0.001), a fixed feeding time (*p* = 0.001), a fixed caregiver (*p* = 0.003), or were usually fed in quiet places (*p* < 0.001) were less likely to have FD.

### Multivariable logistic regression models of the association between FD and transgender-related and family environment characteristics

Three logistic regression models of FD were established. The crude model (model 1) shows the unadjusted logistic regression results of each variable and FD. Model 2 was adjusted for demographic variables that were significantly associated with FD in the univariate analysis. Demographic variables and feeding-related variables that were significantly associated with FD in the univariate analysis were entered into the final model (model 3) to identify transgender-related and family environment factors associated with FD.

As seen in Table 2, before being adjusted, the associated decreased risk factors included having an undergraduate degree (OR = 0.32, 95%CI = 0.17 ∼ 0.57, *p* < 0.001) or postgraduate degree (OR = 0.13, 95%CI = 0.05 ∼ 0.31, *p* < 0.001), with an annual income between 100 ∼ 200k (OR = 0.47, 95%CI = 0.26 ∼ 0.81, *p* < 0.01) or higher than 200k (OR = 0.35, 95%CI = 0.2 ∼ 0.61, *p* < 0.001), feeding their children at fixed time (OR = 0.49, 95%CI = 0.33 ∼ 0.72, *p* < 0.001) or fixed place (OR = 0.48, 95%CI = 0.31 ∼ 0.72, *p* < 0.001), fixed caregiver (OR = 0.44, 95%CI = 0.26 ∼ 0.74, *p* < 0.01), feeding at quiet place (OR = 0.32, 95%CI = 0.2 ∼ 0.52, *p* < 0.001), and having received feeding education (OR = 0.55, 95%CI = 0.34 ∼ 0.89, *p* < 0.05). The associated increased risk factors included letting children eat independently (OR = 1.79, 95%CI = 1.03 ∼ 3.2, *p* < 0.05), coming out after the coming of this child (OR = 2.31, 95%CI = 1.43 ∼ 3.82, *p* < 0.05), family violence (OR = 1.06, 95%CI = 1.04 ∼ 1.08, *p* < 0.001), partner violence (OR = 1.1, 95%CI = 1.07 ∼ 1.14, *p* < 0.001), discrimination during seeking childbearing healthcare (OR = 2.0, 95%CI = 1.37 ∼ 2.93, *p* < 0.001). After being adjusted for socio-demographic variables, the association between eating independently and FD was no longer significant, while the other associations that were significant in model 1 were still significant in model 2. In the final model, the association between coming out after the coming of this child and FD (AOR = 2.26, 95%CI = 1.33 ∼ 3.91, *p* < 0.01) has weakened, but is still significant, whereas the association between feeding education and FD was enhanced (AOR = 0.43, 95%CI = 0.25 ∼ 0.74, *p* < 0.01).

**Table 2.** Multivariable logistic regression models of FD.

	Feeding Difficulties (T score > 70)		
	Model 1	Model 2^a^	Model 3^b^
	OR (95%CI)	AOR (95%CI)	AOR (95%CI)
Age:	1.02(0.98 ∼ 1.05)		
Education level:			
Middle/high/technology/vocational school	1.000 (Ref.)		
College/University (undergraduate)	0.32(0.17 ∼ 0.57)***		
Postgraduate (Master/Ph.D.)	0.13(0.05 ∼ 0.31)***		
Assigned sex:			
Male	1.000 (Ref.)		
Female	0.91(0.62 ∼ 1.33)		
Gender identity:			
Transgender woman	1.000 (Ref.)		
Transgender man	0.92(0.62 ∼ 1.36)		
Family type:			
Extended family	1.000 (Ref.)		
Nuclear Family	0.83(0.55 ∼ 1.25)		
Adoptive/reconstituted family	1.04(0.51 ∼ 2.14)		
Single parent or other	2.02(0.97 ∼ 4.45)		
Annual income:			
<50k	1.000 (Ref.)		
50–100k	0.82(0.44 ∼ 1.52)		
100–200k	0.47(0.26 ∼ 0.81)**		
> =200k	0.35(0.2 ∼ 0.61)***		
Reported gender of child:			
Boy	1.000 (Ref.)		
Girl	0.93(0.63 ∼ 1.38)		
Feeding according to the infant’s hunger signal:			
No	1.000 (Ref.)	1.000 (Ref.)	
Yes	0.71(0.46 ∼ 1.08)	0.78(0.49 ∼ 1.23)	
Verbal praise and emotional communication:			
No	1.000 (Ref.)	1.000 (Ref.)	
Yes	0.76(0.47 ∼ 1.22)	0.81(0.49 ∼ 1.35)	
Fixed feeding place:			
No	1.000 (Ref.)	1.000 (Ref.)	
Yes	0.49(0.33 ∼ 0.72)***	0.58(0.38 ∼ 0.86)**	
Fixed feeding time:			
No	1.000 (Ref.)	1.000 (Ref.)	
Yes	0.48(0.31 ∼ 0.72)***	0.56(0.36 ∼ 0.86)**	
Fixed caregiver:			
No	1.000 (Ref.)	1.000 (Ref.)	
Yes	0.44(0.26 ∼ 0.74)**	0.42(0.24 ∼ 0.72)**	
Feeding at quiet place:			
No	1.000 (Ref.)	1.000 (Ref.)	
Yes	0.32(0.2 ∼ 0.52)***	0.38(0.23 ∼ 0.63)***	
Eating method of child:			
Fed by others	1.000 (Ref.)	1.000 (Ref.)	
Independently	1.79(1.03 ∼ 3.2)*	1.66(0.92 ∼ 3.05)	
Mixed	1(0.65 ∼ 1.55)	1.1(0.7 ∼ 1.74)	
Feeding decision maker:			
Parents	1.000 (Ref.)	1.000 (Ref.)	
Grandparents	1.94(0.93 ∼ 4.36)	1.81(0.83 ∼ 4.19)	
Time of coming out:			
Before the coming of this child	1.000 (Ref.)	1.000 (Ref.)	1.000 (Ref.)
After the coming of this child	2.31(1.43 ∼ 3.82)***	2.45(1.47 ∼ 4.14)***	2.28(1.34 ∼ 3.96)**
Not yet	1.08(0.65 ∼ 1.8)	1.01(0.59 ∼ 1.72)	1.03(0.59 ∼ 1.8)
Hormonotherapy:			
Before pregnancy	1.000 (Ref.)	1.000 (Ref.)	1.000 (Ref.)
After lactation period or never	0.78(0.46 ∼ 1.31)	0.61(0.34 ∼ 1.06)	0.66(0.36 ∼ 1.21)
Gender Dysphoria	1(0.98 ∼ 1.02)	1(0.98 ∼ 1.03)	1(0.98 ∼ 1.03)
Family violence	1.06(1.04 ∼ 1.08)***	1.06(1.04 ∼ 1.09)***	1.06(1.04 ∼ 1.09)***
Partner violence	1.1(1.07 ∼ 1.14)***	1.11(1.07 ∼ 1.14)***	1.11(1.08 ∼ 1.15)***
Family formation method:			
Sexual intercourse	1.000 (Ref.)	1.000 (Ref.)	1.000 (Ref.)
Artificial insemination	1.12(0.58 ∼ 2.23)	1.18(0.59 ∼ 2.41)	1.35(0.64 ∼ 2.92)
Surrogacy	0.64(0.32 ∼ 1.26)	0.8(0.38 ∼ 1.63)	0.89(0.42 ∼ 1.88)
Adoption or other	0.48(0.21 ∼ 1.05)	0.46(0.19 ∼ 1.04)	0.51(0.21 ∼ 1.19)
Feeding Education:			
No	1.000 (Ref.)	1.000 (Ref.)	1.000 (Ref.)
Yes	0.55(0.34 ∼ 0.89)*	0.55(0.33 ∼ 0.91)*	0.43(0.25 ∼ 0.74)**
Discrimination during seeking of childbearing health care:			
No	1.000 (Ref.)	1.000 (Ref.)	1.000 (Ref.)
Yes	2(1.37 ∼ 2.93)***	1.99(1.33 ∼ 3)***	1.99(1.3 ∼ 3.06)**
Primary caregiver:			
Parents	1.000 (Ref.)	1.000 (Ref.)	1.000 (Ref.)
Grandparents	1.07(0.67 ∼ 1.73)	1(0.61 ∼ 1.65)	0.88(0.52 ∼ 1.49)
Relationship with partner:			
Poor	1.000 (Ref.)	1.000 (Ref.)	1.000 (Ref.)
Fair	0.11(0.04 ∼ 0.26)***	0.11(0.04 ∼ 0.25)***	0.09(0.03 ∼ 0.22)***
Good	0.07(0.03 ∼ 0.15)***	0.07(0.02 ∼ 0.16) ***	0.06(0.02 ∼ 0.15)***

AOR, adjusted odds ratio; CI, confidence interval; Ref, reference group. An adjusted odds ratio of 1.00 is the reference. Asterisk(s) denotes significant results (∗*p* < 0.05; ∗∗*p* < 0.01; ∗∗∗*p* < 0.001).

^a^Adjusted odds ratios were adjusted for age and socio-demographic variables which had a significant bivariate association with FD. (Education level, and annual income).

^b^Adjusted odds ratios were adjusted for age and socio-demographic variables and feeding-related variables which had significant bivariate associations with FD. (Education level, annual income, fixed feeding place, fixed feeding time, fixed caregiver, feeding at quiet place).

### Subgroup analysis

**Figure 2.**
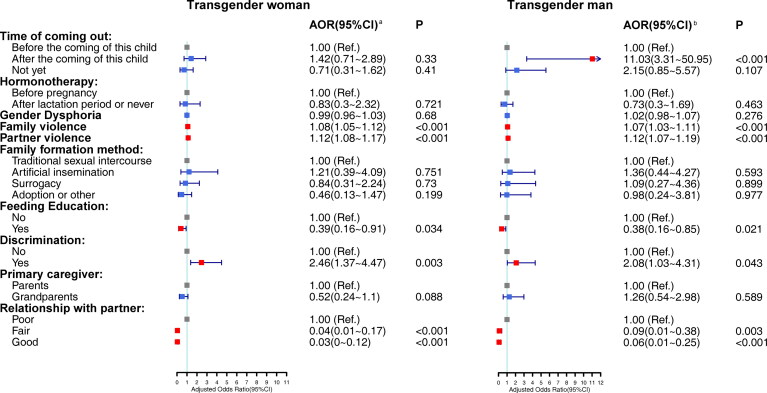
Forest plot of multivariable logistic regression models of FD, and transgender-related characteristics and family environment in transgender woman and transgender man groups. ^a^Odds ratios are adjusted for socio-demographic variables and feeding-related variables which had significant bivariate associations with FD in trans women group (Education level, annual income, family type, feeding according to the infant’ hunger signal, fixed feeding place, fixed feeding time, fixed caregiver, feeding at quiet place). ^b^Odds ratios are adjusted for socio-demographic variables and feeding-related variables which had significant bivariate associations with FD in trans men group. (Education level, fixed feeding place, fixed feeding time, feeding at quiet place).

We used forest plots to visualize the subgroup analysis results of different gender identities. Multivariate logistic regression results for different genders are shown in Figure 2. We only included transgender women and men because the sample size of other gender identities was limited. The association between time of coming out and FD was not significant among transgender women. Other significant associations on FD in all participants were consistent for both men and women.

### Different dimensions of MCH-FS and their correlations with transgender-related and family environment characteristics

**Figure 3.**
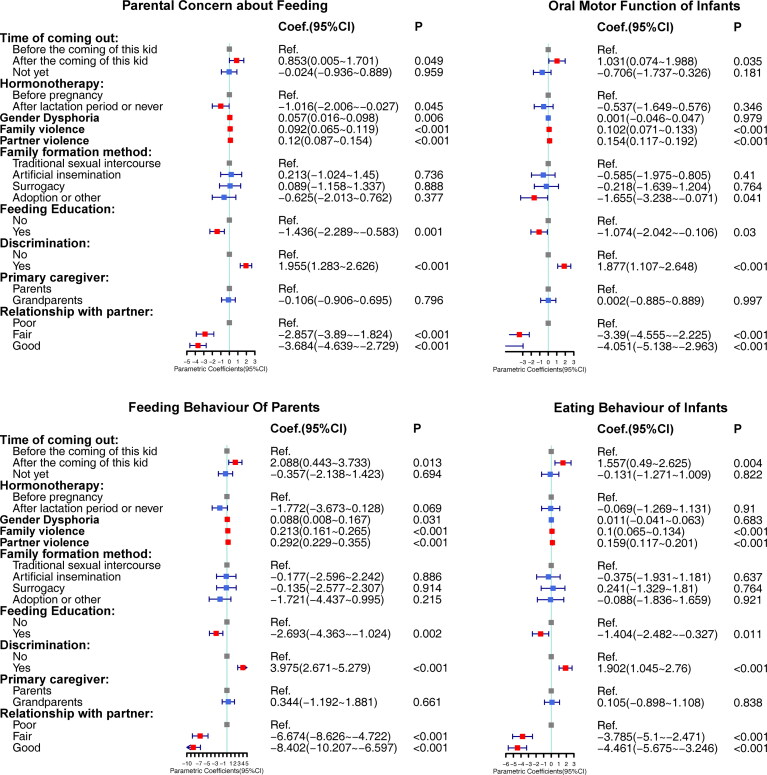
Forest plot of multivariable generalized addictive models of FD, and transgender-related characteristics and family environment, in four dimensions. ^a^Coefficients are adjusted for socio-demographic variables and feeding-related variables which had significant bivariate associations with parental concern about feeding (Education level, annual income, fixed feeding place, fixed caregiver, feeding at quiet place, feeding method). ^b^Coefficients are adjusted for socio-demographic variables and feeding-related variables which had significant bivariate associations with oral motor function of infant (Education level, annual income, feeding according to the infant’s hunger signal, verbal praise and emotional communication, fixed feeding place, fixed feeding time, fixed caregiver, feeding at quiet place, and feeding method). ^c^Coefficients are adjusted for socio-demographic variables and feeding-related variables which had significant bivariate associations with feeding behavior of parents (Education level, annual income, feeding according to the infant’s hunger signal, fixed feeding place, fixed feeding time, fixed caregiver, feeding at quiet place, and feeding method). ^d^Coefficients are adjusted for socio-demographic variables and feeding-related variables which had significant bivariate associations with eating behavior of infants (Education level, annual income, feeding according to the infant’s hunger signal, verbal praise and emotional communication, fixed feeding place, fixed feeding time, fixed caregiver, feeding at quiet place, and feeding method).

Figure 3 demonstrated the results of generalized additive models for different dimensions of MCH-FS: PCF, OMFI, FBP, and EBI. All the significant effects on FD were consistent with those on OMFI, FBP, and EBI, including the effects of time of coming out, family violence, partner violence, feeding education, discrimination during the seeking of childbearing, and relationship with partner. The effect of time of coming out was not found in PCF, whereas taking hormonotherapy after the lactation period or never was found to be negatively correlated with PCF. In addition, a negative correlation between adoption and OMFI, positive correlations between gender dysphoria and FBP, and gender dysphoria and PCF were also observed.

### Pathway analysis

**Figure 4.**
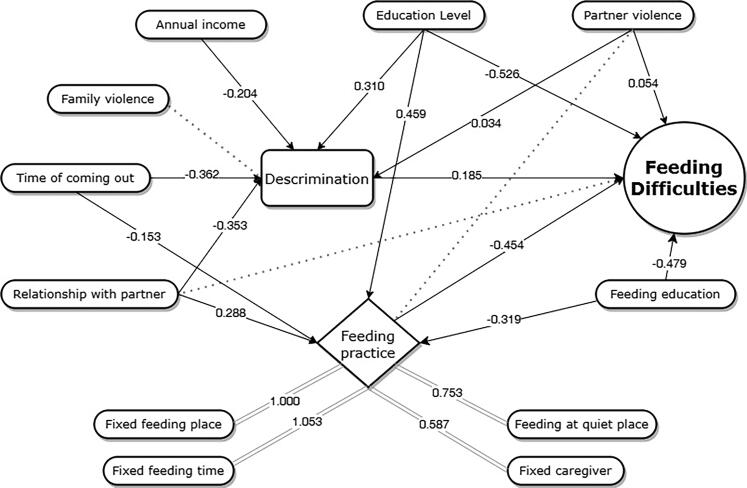
The pathway analysis on feeding difficulties.

The principle of establishing SEM involves variables in the regression analyses that are significantly relevant to FD. Feeding practice is a latent variable measured by a fixed feeding place, a fixed feeding time, feeding at a quiet place, and a fixed caregiver. Figure 4 shows the pathway analysis of FD. The final SEM for FD indicated a good fit: root mean square error of approximation (RMSEA) = 0.035, comparative fit index (CFI) = 0.953, Tucker-Lewis index (TLI) = 0.927, and weighted root mean square residual (WRMR) = 0.900. The specific standardized effect indicated that feeding practice was negatively associated with FD. Higher education level and poor relationship with partner were positively correlated with feeding practice, while early time of coming out and poor feeding education were negatively correlated with feeding practice. A positive association between discrimination and FD was also observed. Higher annual income level, late time of coming out, and a better relationship with partner were negatively correlated with discrimination, while lower education levels and partner violence were positively associated with discrimination. Moreover, the standardized indirect effects of education level, feeding education, and relationship with partner on FD were significant, with pathway coefficient values of −0.151, 0.145, and −0.196, respectively.

## Discussion

Using a large-scale cross-sectional survey, this study revealed the FD situation among children of transgender parents in China. In our study, more than half of (55.4%) children could be categorized as having FD, and 34.5% were recognized as having severe FD, which is higher than it in preschool children from the general population (ranges from 14 to 23%, 3% to 10% are reported to be severe to general populations) (Ramsay et al., 2011; Ren et al., 2021; Rogers et al., 2018). These findings indicated that children in transgender families may suffer more common and severe FD, and relevant influencing factors must urgently be explored.

First, our study found that some common sociodemographic factors may also influence feeding experiences in transgender families. Consistent with findings in general populations, the higher the parent’s educational level, the less likely the child is to develop FD (Ramsay et al., 2011). We also found that the lower the family annual income, the more likely the child is to develop FD, which is not a significant contributor to FD in cisgender families (Evans et al., 2011; Maranhão et al., 2017; Ren et al., 2021). This phenomenon could be explained by the influence of family income on social status. Our pathway analysis also demonstrated that family income can indirectly influence FD through discrimination. A large survey of transgender adults in the United States showed that having low income and experiences of discrimination during health care are positively associated with less access to healthcare (White Hughto et al., 2016), which supports our results.

Besides, we found that some feeding-related factors may be associated with children’s FD in transgender families. Previously, many studies have identified the importance of early feeding behaviours and a high-quality food environment (Rogers et al., 2018). Parents’ feeding habits have been shown to be closely related to their children’s eating behaviour (Ek et al., 2016), where a literature review suggested that parents’ eating habits and feeding strategies were the most important determinants of children’s eating behaviour and food choices (Scaglioni et al., 2018). Our study also revealed certain associations between feeding behaviour and environment, and FD. The analysis showed that providing a fixed feeding place, a fixed feeding time, a fixed caregiver, and feeding children at a quiet place are all favourable feeding strategies.

Furthermore, according to the pathway analysis results, receiving feeding education is also a protective factor that can reduce FD by improving feeding behaviours. However, due to the prevalence of transphobia in the social environment (Hafford-Letchfield et al., 2019), it is hard for transgender individuals to get enough professional help (Hoffkling et al., 2017). Our previous study also revealed that the proportion of transgender people who have received feeding education (76.7%) needs to be improved, and it is significantly associated with breastfeeding and chest feeding practice (Yang et al., 2023). Besides, less than half (43.4%) of participants in our study reported that they received feeding knowledge from professional healthcare providers, the main source of their parenting knowledge is the social media, which may lead to inappropriate feeding behaviours.

In addition to sociodemographic factors and feeding behaviours, we found that some unique characteristics related to transgender populations contributed to FD in their children. Disclosure, or “coming out”, refers to the way in which LGBTQ + individuals reveal their gender identity or sexual orientation and is an important process in LGBTQ + families that has received increased attention in recent years (Hunter, 2007). Available data suggests that initial disclosure is an important part of transgender identity development and a stressful time for transgender parents (Veldorale-Griffin & Darling, 2016). Previous quantitative research has shown that children in transgender families may face difficulties adapting to a range of serious life events, including parental separation or divorce, considerable family conflict, etc., while their parents are going through gender transition periods (White & Ettner, 2007). Children with this experience also show higher rates of eating disorders (White & Ettner, 2007). Similarly, our study found that transgender parents coming out after having children was positively correlated with their children’s FD, because coming out may bring about a series of changes and even conflicts in the family environment.

Pathway analysis shows that coming out indirectly affects FD through discrimination and feeding practices. However, after subgroup analysis stratified for transgender women and men, this association was only significant in transgender men. Before coming out to their families, transgender men may present as cisgender women in their family and assume the role of "mother" to avoid more discrimination and possible transphobic violence (Hoffkling et al., 2017). In previous studies, feeding problems tended to be more closely related to the behaviour and characteristics of mothers, who are usually the ones who take more care of their children, especially in Chinese families (Guo et al., 2022). However, coming out after having a child must bring a shift in roles, which may have an impact on family relationships, and children may need to adjust to new parenthood, leading to the possibility of FD. It should not be overlooked that there was also some participants who reported other gender identities, and situations of these populations should be further investigated in the future, with larger sample size.

As mentioned above, family environment is an important factor affecting parenting. Apart from the effects of coming out, we also found that children with transgender parents who had suffered from FV and PV were more likely to experience FD. More negative home environments (high family conflict, low family cohesion, and increased household chaos) have been found to be associated with more unhealthy food-related behaviours (Iwinski et al., 2021). However, traditional values of the Chinese family unit may result in the exclusion and violence of transgender individuals, and notions such as observance of "rules of nature" and “perpetuation of blood for families” make gender and sexual minorities a disgrace to the family (Wang et al., 2020). According to one study, 92.8% of transgender youth in China have experienced parental abuse or neglect because of their gender identity (Peng et al., 2019).

Meanwhile, the better the relationship with the partner, the lower the probability of the child’s FD. Family functioning is considered especially important for transgender parents, who often experience some form of family exclusion due to gender transition (Veldorale-Griffin & Darling, 2016). Family acceptance has been shown to be a protective factor against many negative outcomes for transgender people, including substance abuse and suicide attempts (Carone et al., 2021). Transgender individuals are more likely to experience various forms of violence than cisgender individuals (Harden et al., 2022). And it might be one of the reasons for such a high FD rate observed in our stydt. Freedman et al., 2002 found that family conflict is an important risk factor affecting children. Of note, studies have demonstrated that it is not the identity transition itself that affects transgender parenthood, but the parental conflict and relationship breakdown that can impact it (Veldorale-Griffin, 2014). The response and support of partners of the transgender individuals can also affect their children’s experience (Hines, 2006). Our findings and previous evidence advocated that family support and family function are particularly important for reducing the FD rate among children of transgender families; having a good family environment might address this problem from the root.

It should be highlighted that, as the results of pathway analysis show, many factors can indirectly affect feeding difficulties in children among transgender families through discrimination during the seeking of childbearing health care. Discrimination is an important issue in LGBTQ-related research. Studies have found that transgender individuals face much higher rates of health risks, unemployment, homelessness, job discrimination, and workplace harassment compared to the general population (Veldorale-Griffin & Darling, 2016). Even, transgender people are more likely than cisgender gay or lesbian people to suffer serious physical harm caused by discrimination, including hospitalization and death (De Castro-Peraza et al., 2019; Hoffkling et al., 2017; Kuehnle & Sullivan, 2001).

This discrimination is also evident in the health system, where transgender individuals face high levels of inequality in access to healthcare services(Bockting et al., 2013). In the medical context, the stigma of transgender collectives can lead to inadequate information provided by health professionals and the display of intense microaggression (De Castro-Peraza et al., 2019). This behaviour, in turn, can cause transgender people to avoid seeking medical attention or hide relevant personal information (Kuehnle & Sullivan, 2001). Invisibility, transphobia, and violence create barriers to transgender individuals accessing the appropriate medical resources they need (De Castro-Peraza et al., 2019). Diversity education therefore appears to be a core need for training health professionals, who have a responsibility to fully support transgender people in choosing to raise their children and provide them with necessary feeding advice.

Interestingly, we also have some intriguing findings from our multidimensional analysis of MCH-FS. First of all, significant factors in the analysis of total MCH-FS are basically consistent in the analysis of four dimensions. This suggests that these factors affect FD formation from all aspects. Besides, gender dysphoria was found to have positive correlations with parental concern about feeding and the feeding behaviour of parents. This phenomenon can be explained by two theories. Transgender individuals with severe gender dysphoria may have experienced more negative, pathological, and demeaning opinions from others, thus needing to prove their ability and adequacy to fulfill their role as parents and then showing more concern for their children (McGuire et al., 2016). Meanwhile, gender dysphoria usually links with more shame; Veldorale-Griffin and Darling have suggested that stigma is associated with more stressful and negative family interactions, thus affecting the feeding behaviour and health-seeking behaviours of transgender parents.

In addition, associations between hormonotherapy and PCF, and adoption and OMFI, were also found. According to the current consensus, the use of hormone therapy may affect infant health; for example, testosterone has been found to be at teratogenic risk (García-Acosta et al., 2019). Therefore, it is reasonable to infer that transgender parents who have not received hormone therapy before having children show less concern about feeding. Another finding, adopted children may have fewer disorders of oral motor function, currently lacks evidence and theory to be supported. Transgender families come in more diverse forms, and in addition to biological pregnancy, adoption is increasingly becoming a common choice among the transgender population (Goldberg, 2023). Further research is needed to determine the effects of adoption on children and the reasons for it.

Our study boasts several advantages. Firstly, this is the first large-scale survey that quantitatively investigated the prevalence of FD among children of transgender families in China. Secondly, this study explores the multiple factors associated with FD, including individual factors, social factors, and family circumstances. Most importantly, it points out the direction for how to provide medical and social support to transgender parents. However, the study also has some limitations. First, due to the nature of the cross-sectional design, this study cannot adequately examine the causal relationship between the variables. Second, with regard to sampling methods, current strategies are the most effective way to recruit gender and sexual minorities; however, the representativeness is compromised, selection bias, non-response bias, and other unknown biases may exist. In addition, the data collection through electronic questionnaires can also cause self-reporting bias. Some transgender parents who are older or living in rural areas without access to the internet or transgender communities may not have been included in the study. Third, the study assessed children through the parents’ reports and did not gather information from the perspective of the child or a healthcare professional, thus not being able to explore children’s internalization disorders. The composition and family environment of transgender families may more complex than those of the general population, involving many factors specific to this population. Fourth, because of the lack of prior studies, there was no basis for calculating sample sizes for subgroup analyses, the sample sizes for the different gender groups in the subgroup analyses might be insufficient, especially for the participants who reported their gender as other, including “gender queer”, “non-binary”, and so on. The factors associated with FD may differ in different population. Therefore, long-term follow-up studies in children of transgender family are needed to investigate potential health concerns and their underlying causes, ensuring their optimal growth and development.

## Conclusion

This study shows that the FD rate among children of Chinese transgender families might be an issue of concern. In addition to common social determinants associated with FD, certain influencing factors were observed in this population, including feeding behaviours, poor family environments, and discrimination when seeking healthcare. This study advocates a comprehensive effort to improve parenthood in transgender populations, including: (1) providing more family support to transgender individuals; (2) developing specific feeding education strategies for transgender parents; and (3) eliminating discrimination in the health system. Most importantly, more research is needed to understand the parenting issues faced by gender minorities and to provide a better upbringing for children and adolescents.

## Data Availability

The data that support the findings of this study are available on request from the corresponding authors. The data are not publicly available due to privacy or ethical restrictions.

## List of abbreviations

FD: Feeding difficulties
SEM: Structural equation modeling
MCH-FS: The Montreal Children’s Hospital Feeding Scale
PCF: Parental concern about feeding
OMFI: Oral motor function of infants
FBP: Feeding behaviour of parents
EBI: Eating behaviour of infants
WLSMV: Weighted least-squares mean- and variance-adjusted
OR: Odds ratio
AOR: Adjusted odds ratio
CI: Confidence interval
